# Co-Occurrence of Asthma and Nephrolithiasis in Children

**DOI:** 10.1371/journal.pone.0168813

**Published:** 2017-01-12

**Authors:** Ganesh K. Kartha, Ina Li, Suzy Comhair, Serpil C. Erzurum, Manoj Monga

**Affiliations:** 1 Glickman Urological and Kidney Institute, Cleveland Clinic, Cleveland, OH, United States of America; 2 Respiratory Institute and Lerner Research Institute, Cleveland Clinic, Cleveland, OH, United States of America; University of Nottingham, UNITED KINGDOM

## Abstract

It has been proposed that epithelial dysfunction and inflammation may predispose patients to kidney stone formation. Asthma is another chronic condition related to epithelial dysfunction and inflammation. We hypothesized that pediatric patients with asthma would have an increased prevalence of nephrolithiasis. Furthermore, we investigated if asthma patients with nephrolithiasis have clinical characteristics and urine profiles that point to mechanisms of stone formation. We evaluated 865 pediatric patients who had a diagnosis of nephrolithiasis. Clinical/demographic data and 24 hour urine samples were compared between asthma + stone (n = 142) and stone only patients. Data from asthmatics without stone were also available for evaluation of medication differences among asthma + stone and asthma only patients. The prevalence of nephrolithiasis in the pediatric population at our institution was 0.08% vs. 0.31% in our pediatric asthmatic population. The prevalence of asthma in our pediatric population was 6.8% vs. 26.7% in our pediatric stone patients. Asthma + stone patients were more likely to be on a combination inhaled corticosteroid + long acting beta agonist inhaler as compared to age/gender/BMI matched asthma patients without stone (29.7% vs. 13.7%, p = 0.0012). 259 kidney stone patients had 24 hour urine samples for comparison. There was no difference in 24 hour urine profiles between asthma + stone and stone only patients. Children with asthma have a 4-fold greater prevalence of kidney stones than the general pediatric population. Similarly, children with kidney stones have a 4-fold greater prevalence of asthma. This correlation may suggest a mechanistic link between asthma and nephrolithiasis. Further investigation is needed to elucidate the pathophysiologic origin of this relationship.

## Introduction

Kidney stones in children are rare; however the incidence seems to be increasing [1]. Children with stones are at an increased risk of developing recurrent stones, and thus metabolic workup for identifying underlying pathophysiological processes are recommended. Risk factors that contribute to stone formation in children include metabolic abnormalities and/or anatomical variations in the urinary tract that may lead to abnormal urine composition, urinary stasis and/or urinary tract infections. A study from Mayo Clinic revealed that 52% of pediatric patients with kidney stones had an underlying metabolic or anatomical etiology for nephrolithiasis [1, 2]. In addition to metabolic and structural abnormalities, associated co-morbid conditions such as obesity have also been linked to pediatric stone formation. One theory is the increased prevalence in obesity may be contributing to the increased prevalence in pediatric nephrolithiasis [2, 3]. It is currently unclear if other childhood co-morbid conditions are associated with pediatric nephrolithiasis.

Asthma is the most common chronic disease of childhood; affecting 8.3% of the pediatric population according to the Center for Disease Control. Asthma is characterized by alterations of epithelial ion channel activation in the lung which is classically induced by inflammatory reaction such as to an allergen [4]. Urinary epithelium ion channel abnormalities related to inflammation are mechanistically linked to kidney stone formation [5]. In the context that stones and asthma are both characterized by inflammatory epithelial disorders, we hypothesized that children with asthma might have a higher prevalence of stones and that stone formation, with underlying epithelial defects, might lead to a propensity of more asthma.

## Material and Methods

This study was performed via extraction from our electronic medical records under an International Review Board approved study. The approval was from the Cleveland Clinic International Review Board and the data were analyzed anonymously.

For overall prevalence (as of April, 2015), pediatric asthma and kidney stone patients included were identified via ICD-9 diagnosis codes (Asthma diagnosis: 471.9, 493, 493.01, 493.02, 493.1, 493.11, 493.12, 493.2, 493.21, 493.22, 493.8, 493.81, 493.82, 493.9, 493.91, 493.92, 495.8, 500, 504, 506.3, 506.9, 507.8, 516.9, 518.3: Stone diagnosis 592.0, 592.1, 592.9, 594, 594.1, 594.2, 594.8, 594.9).

Data for comparative analysis was gathered on patients with a kidney stone diagnosis at our institution between January 2000 and September 2014. Patients included were required to be 6 months to 18 years old at the time of stone diagnosis. Asthma diagnosis, medications, age, gender, race and BMI were obtained from the electronic medical record. A BMI, age, gender matched cohort of asthma only patients was extracted for comparative analysis. The 24 hour urine profiles were performed for clinical indication and available for study analyses in those with stones. All 24 hour urines were performed using an outsourced company or our internal stone panel analysis. The first 24 hour urine sample after stone diagnosis was used for analysis in all cases per clinical standard of care. The 24 hour urinary volumes, sodium, calcium, citrate, oxalate and creatinine were compared. All values were controlled by creatinine levels. Patients missing any data points after chart review were excluded from the study

Statistical analysis was performed using the JMP 10.0 statistical software. Categorical variables were compared with Fisher’s exact test. Numerical variables were compared with student t-test. P value was set at < 0.05 for significance.

## Results

**Fig 1:**
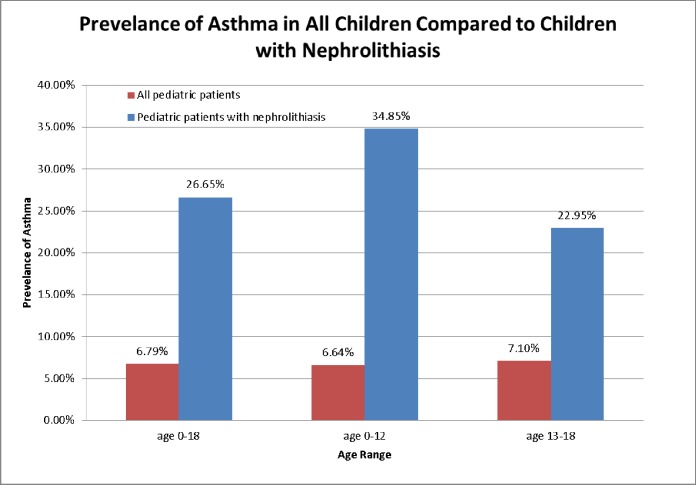
The prevalence of asthma at our institution in all children and children with nephrolithiasis.

**Fig 2:**
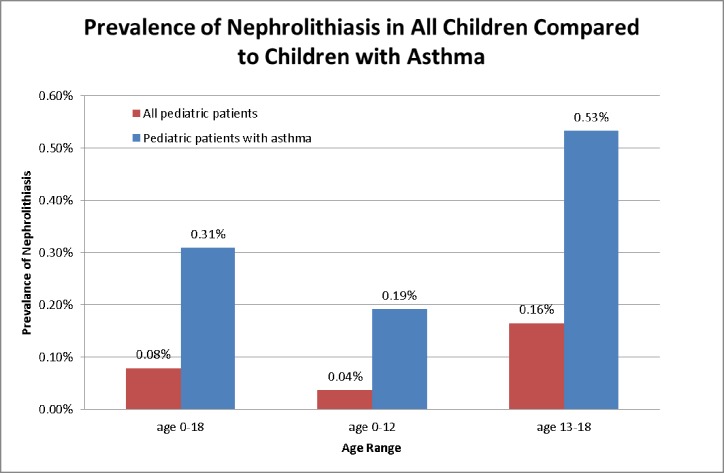
The prevalence of nephrolithiasis at our institution in all children and children with asthma.

As of April 2015, there were 538, 001 pediatric patients that had received care in our network. The general prevalence of nephrolithiasis in the pediatric population was 0.08%, however it was 0.31% in our pediatric asthmatic population, and specifically 0.53% in our adolescent (13–18 years old) asthmatic population. The prevalence of asthma in our pediatric population was 6.79%, however it was 26.65% in our pediatric stone patients and 34.85% in pediatric stone patients younger than 12. [Figs 1 and 2] shows the prevalence of asthma and kidney stones in the general pediatric population, the prevalence of kidney stones in our asthma pediatric population, and the prevalence of asthma in our pediatric stone population. [Table 1] depicts the demographic data for current pediatric patients stratified by asthma and stone diagnosis.

**Table 1:** Clinical-Demographic data for current pediatric patients stratified by asthma and kidney stone diagnosis.

	All Pediatric Patients (n = 538001)	Pediatric Patients with Asthma (n = 36518)	Pediatric Patients with Nephrolithiasis (n = 424)	Pediatric Patients with Asthma and Nephrolithiasis (n = 113)
**% male**	51.8%	59.3%	51.4%	49.5%
**% White**	63.1%	69.2%	83.6%	82.9%
**% African American**	18.4%	19.7%	4.5%	4.5%
**% Hispanic**	1.16%	0.47%	0.95%	0.0%
**BMI**	21.6	18.6	21.3	21.9

We identified 865 patients between January 2000 and September 2014 that were given a nephrolithiasis diagnosis at an age 6 months to 18 years with all available data. Of these 865 patients, 142 had a diagnosis of asthma. Clinical demographic information stratified by diagnosis of asthma for all pediatric stone patients during this time period is listed in [Table 2]. Stone patients with asthma tended to be male (51.06% vs. 41.99%, p = 0.0476) and have a stones at a younger age (12.33 vs. 13.51 years old, p = 0.0038) compared to stone patients without a diagnosis of asthma. There was no difference in BMI between asthma-stone patients and stone only patients at the time of stone diagnosis (22.62 vs. 21.86, p = 0.1875).

**Table 2:** Clinical-demographic data comparison between asthma-stone patients and stone patients without asthma.

	Children with Nephrolithiasis Only (n = 723)	Children with Asthma and Nephrolithiasis (n = 142)	p value
**Age at Nephrolithiasis diagnosis (Mean +/- SD)**	13.5 ± 4.4	12.3 ± 4.7	0.003
**BMI at Nephrolithiasis Diagnosis (Mean +/- SD)**	21.9 ± 6.2	22.6 ± 6.7	0.18
**% Male**	42%	51%	0.047

Medication profiles between asthma-stone patients and age/gender/BMI matched asthma only patients were compared in [Table 3]. Asthma-stone patients were more likely to be on a combination inhaled corticosteroid + long acting beta agonist than asthma patients without stone (29.7% vs. 13.7%, p = 0.0012). There was no difference in inhaled beta agonists (94.5% vs. 95.2%, p = 0.8004), inhaled corticosteroid (44.5% vs. 50.7%, p = 0.3088), or any anti-asthmatic medication (96.9% vs. 96.6%, p = 0.8894) use between asthma patients with or without a stone diagnosis.

**Table 3:** Prescribed asthma medication comparison between asthma patients with and without kidney stones matched by age gender and BMI.

Medications	Children with Asthma and Nephrolithiasis (n = 142)	Matched Children with Asthma Only (n = 142)	p value
**% prescribed anti asthmatic medication**	96.9	96.6	0.89
**% prescribed inhaled corticosteroids**	44.5	50.7	0.31
**% prescribed inhaled beta-agonists**	94.5	95.2	0.8
**% prescribed inhaled combination corticosteroid/beta agonist**	29.7	13.7	0.001

259 kidney stone patients in our cohort had available 24 hour urine samples for comparison [Table 4]. There was no difference in 24 hour urine profiles between asthma-stone patients and stone only patients when adjusted for creatinine.

**Table 4:** 24 hour urine comparison between asthma patients with stone and those with stone only diagnosis (Cr = Creatinine, SD = Standard deviation).

**24hr Urine Values** Mean; (SD)	**Normal Range**	**Asthma + Stone (n = 51)**	**Stone only (n = 208)**	**p value**
Cr(mg)/day	—	1011.9 ± 582.3	1001.7 ± 511.6	0.9
Weight (kg)	—	47.4 ± 21.1	48.4 ± 24.3	0.79
Calcium (mg/day)	< 250	160.6 ± 109.2	163.6 ± 100.3	0.86
Citrate (mg/day)	> 450	427.2 ± 308.2	409.9 ± 264.5	0.73
Oxalate (mg/day)	20–40	39.0 ± 21.6	38.7 ± 21.3	0.93
Sodium (mg/day)	50–150	149.8 ± 101.7	129.4 ± 74.5	0.17
Uric acid (mg/day)	< 800	399.6 ± 204.0	415.2 ± 217.7	0.69
Volume (ml)/day	—	1147.5 ± 681.8	1297.4 ± 829.9	0.36
Serum creatinine (mg/dl)	—	0.7	0.66	0.4
**24hr Urine Values/Cr**		**Asthma + Stone (n = 51)**	**Stone only (n = 208)**	**p value**
Calcium/Cr	—	0.19 ± 0.19	0.18 ± 0.01	0.67
Citrate /Cr	—	0.53 ± 0.25	0.48 ± 0.30	0.32
Oxalate/Cr	—	0.05 ± 0.06	0.04 ± 0.02	0.32
Sodium/Cr	—	0.16± 0.09	0.14 ± 0.08	0.26
Uric acid/Cr	—	0.5 ± 0.23	0.46 ± 0.19	0.28

## Discussion

In this study, we identify a co-occurrence of asthma and nephrolithiasis in children. To our knowledge, this is the first report of an association between asthma and kidney stone formation. Asthma prevalence has been increasing in the past 20 years. Likewise, although the prevalence of kidney stones is rare in the pediatric population, the rate is rising. Pediatric stone formation has been linked to metabolic/genetic abnormalities, including obesity and cystic fibrosis. In cystic fibrosis (CF), the mechanism of greater kidney stone is related to the epithelial ion channel dysfunction. The CFTR (cystic fibrosis transmembrane conductance regulator) mutation affects chloride ion channels causing known abnormalities in the respiratory epithelial secretions. It has been hypothesized that similar electrolyte handling in the renal epithelium may predispose CF patients to kidney stone formation [6]. Patients with CF typically get stones during adolescence or early adulthood. The prevalence of nephrolithiasis in CF adolescent patients is 4–6% which is significantly higher than the prevalence of kidney stone formation in the general adolescent population or in our population of children with asthma^6^. The greater prevalence of kidney stones in CF and asthma supports a link between airway inflammatory disease and renal tubular handling of electrolytes and stone formation. By definition, asthma is a chronic inflammatory condition that affects the epithelial lining of the lungs. Recent studies have suggested inflammatory processes in the kidney may lead to stone formation [5, 7, 8]. Inflammation plays a role in altering the glycosaminoglycan composition in the epithelial lining of the kidney, leading to increased crystallization and/or rupture of Randall’s plaques (interstitial renal calcifications) from renal papillary epithelium. Similarly, studies have shown that inflammatory conditions and reactive oxygen species play a role in glycosaminoglycan degradation in respiratory epithelium [9]. Independently, nephrolithiasis has been associated with alterations in the glycosaminoglycan layer of the urothelium and asthma has been associated with changes in the glycosaminoglycan layer in the lung epithelium. To our knowledge, there have been no investigations looking at inflammatory changes in the urothelium in asthma patients.

Here, we also sought to identify possible mechanisms for the concordance of asthma and stones. First, we compared clinical demographic data, specifically BMI, to see if this could be causing a difference in stone formation between stone only and stone patients with asthma. However, BMI of individuals was similar among stone patients with asthma and those without. Asthma-stone patients were prescribed combined inhaled corticosteroid and long acting beta agonist more than age/gender/BMI matched asthma only patients. There is an association of glucocorticoid use with increased bone resorption leading to nephrolithiasis in neonates [10, 11], however overall corticosteroid use in our cohort was no different between asthma stone patients and those with asthma only. A higher rate of combination inhaled medication, which is prescribed in a stepped-care manner for asthma care, was found in those asthmatics with stones, suggesting that those individuals with more severe asthma had stones. Unfortunately, pulmonary function testing is not routinely performed in children with asthma, as airflow is usually well preserved in children unless there is very severe asthma. Thus, pulmonary function tests were not available for most of the cohort which had mild to moderate asthma. Notably, our asthma only group did not routinely undergo imaging studies; it is feasible that some of these patients may indeed have asymptomatic renal stones; however this would only strengthen the association identified in this study.

Other confounding variables that may contribute to this correlation maybe socioeconomic factors and other similar underlying concurrent diagnoses. Multiple prior studies have suggested that certain socioeconomic factors may predispose children to kidney stones as well as asthma [12, 13]. However, socioeconomic data was not available for this study and is a limitation of our dataset. An additional limitation is that our data represents general pediatric patients seen in a tertiary referral center which may not represent the general population as a whole. Patients seen at our institution may have more comorbidities and chronic diseases that may contribute to both asthma and/or nephrolithiasis. Furthermore, our patient population is predominantly white. Our observations may not be consistent amongst other racial groups. Further investigation is needed to elucidate whether other possible concurrent diagnoses and clinical/demographic characteristics may contribute to our findings.

As CF patients have epithelial ion channel abnormalities in both the lung and kidney, we investigated urinary profiles of asthma patients (who have epithelial abnormalities of the lung) to see if similar urothelial abnormalities maybe seen with urinary electrolyte handling. To investigate if there were underlying abnormalities in asthmatics with stones that were different from stone only children, we evaluated 24 hour urine profiles. Hypercalciuria is the most common lithogenic risk factor seen in children with kidney stones [14–16]. In children with stone disease associated with a metabolic abnormality, hypercalciuria can be seen in up to 50% of patients [17]. Another leading cause of stone formation in the pediatric population is hypocitraturia, which been seen in 10–60% of pediatric patients with calcium renal calculi [18–20]. Urinary citrate is an inhibitor to calcium stone formations and crystallization via calcium binding making it more soluble. Low levels of citrate in the urine can lead to nephrolithiasis. Chronic metabolic acidosis can increase the risk of nephrolithiasis by increasing kidney tubule citrate reabsorption causing hypocitraturia. In our cohort, we did notice a low 24 hour urine level of citrate in both stone patients and stone patients with asthma. However, the absolute value and creatinine adjusted difference between the groups was not significantly different between stone patients with and without asthma. Overall, the 24 hour urine profiles of stone patients with and without asthma were similar, suggesting that asthma patients do not have different urine concentrating/solute handling abnormalities when compared to non-asthma stone patients.

## Conclusion

Children with asthma have a 4-fold greater prevalence of kidney stones than the general pediatric population at our institution. Similarly, children with kidney stones have a 4-fold greater prevalence of asthma, such that 1 in 3 children with stones under the age of 12 have asthma. Although this is a correlation study, the data may suggests a mechanistic link between asthma and nephrolithiasis. Further investigation is needed to elucidate the pathophysiologic origin of this relationship.
